# Flexible Conductive Cellulose Network-Based Composite Hydrogel for Multifunctional Supercapacitors

**DOI:** 10.3390/polym12061369

**Published:** 2020-06-18

**Authors:** Shaoqiu Ke, Zhiqi Wang, Kai Zhang, Fangchao Cheng, Jianping Sun, Nannan Wang, Yanqiu Zhu

**Affiliations:** 1 Guangxi Key Laboratory of Processing for Nonferrous Metallic and Featured Materials, School of Resources, Environment and Materials, Guangxi University, Nanning 530004, China; shaoqiuke@126.com (S.K.); zhiqiwang@st.gxu.edu.cn (Z.W.); zhangkai981219@163.com (K.Z.); jpsun@gxu.edu.cn (J.S.); 2 College of Material Science and Engineering, Central South University of Forestry and Technology, Changsha 410004, China; 3 GIFT (Guangxi Institute for Fullerene Technology), School of Resources, Environment and Materials, Guangxi University, Guangxi 530004, China; 4 College of Engineering, Mathematics and Physical Sciences, University of Exeter, Exeter EX4 4SB, UK

**Keywords:** cellulose network, polyaniline, electrochromic supercapacitor, integrated structure

## Abstract

With the continuous development of energy storage devices towards sustainability and versatility, the development of biomass-based multi-functional energy storage devices has become one of the important directions. In this study, a symmetric dual-function supercapacitor was constructed based on a cellulose network/polyacrylamide/polyaniline (CPP) composite hydrogel. The presented supercapacitor, with excellent electrochemical performance and an areal capacitance of 1.73 mF/cm^2^ at 5 mV/s, an energy density of 0.62 µW h/cm^2^ at a power density of 7.03 µW/cm^2^, a wide electrochemical window of 1.6 V and a promising cycling stability, can be achieved. The transmittance of the supercapacitor at 500 nm decreased by 9.6% after the electrification at 3 V, and the device can exhibit periodic transmittance change under the square potential input between 0.0 V and 3.0 V at regular intervals of 10 s. The present construction strategy provides a basis for the preparation of multifunctional devices with natural renewable materials and structures.

## 1. Introduction

The increasing energy demand and the outbreak of environmental crises drive human beings to pursue environmentally friendly, multifunctional energy storage materials and multifunctional energy storage devices [1]. As an energy storage device with an optical function, electrochromic supercapacitors (ESCs) have attracted much attention because of their advantages, such as rapid color change and efficient energy storage, while meeting specific application needs, such as smart windows, electronic paper, and auto-dimmer rearview mirrors [2,3,4]. Current electrochromic materials mainly include transition metal oxides (such as WO_3_ and V_2_O_5_) [5,6,7], Prussian blue, and conductive polymers (such as polyaniline (PANI) and polythiophene) [3]. Among these, tungsten trioxide (WO_3_) serves as a cathodically coloring material, with PANI as an anodically coloring material, and there is a simultaneous complementary coloration for both of them in the electrochromic application. WO_3_ is generally taken as the negative electrode and PANI as the positive electrode to study the asymmetric electrochromic supercapacitor [8,9,10,11]. In particular, PANI has shown excellent compatibility with cellulosic materials (e.g., cellulose networks) to prepare novel electrode materials and biomass-based energy storage devices [12].

The structure of electrochromic devices is similar to that of supercapacitors, so the structure of electrochromic supercapacitors includes the structure of these two devices, generally including a transparent conductive layer, electrochromic layer, electrolyte layer, and ion storage layer [13]. In the whole device, the electrochromic layer is responsible for the color change with the change in the electric field. Its performance is mainly determined by the electrode material, but, in addition to the electrode material, the electrolyte and the structure of the device also play an important role in the ESC performance. The intermediate electrolyte layer is usually composed of a clear liquid or solid electrolyte, which is usually used in combination with a solid gel to prevent electrolyte leakage. Due to its advantages of high transparency and low cost, polyacrylamide (PAM) can be combined with lithium salt, sodium salt, and potassium salt to form conductive transparent gel electrolytes [14].

The structural design of ESC is of great significance for making full use of electrode materials, promoting the transport of ions and electrons, and enabling the device to have a good optical modulation performance. Traditional supercapacitors and electrochromic devices are composed of multilayer structures assembled layer-by-layer, which, due to the interaction between multiple interfaces, hinder the rapid transfer of charges and ions [15]. An integrated structure can be introduced to reduce the number of layers of ESC and the contact resistance between layers, so as to achieve high-performance energy storage and optical modulation performance. Electrochemical deposition is usually carried out to construct an integrated structure based on gel electrolytes, while the deposition process will influence the flexibility and mechanical properties of gel electrolytes. Therefore, a flexible, transparent, robust material is needed to enhance the gel electrolytes, such as cellulose [16,17]. Based on the above discussion, an integrated ESC is expected to be constructed by combining a gel electrolyte with cellulose and forming the integrated structure through the electrochemical deposition of the active substance.

To verify this hypothesis, a new type of ESC was developed based on a cellulose network. In the structure of ESC, the cellulose network/PAM/PANI (CPP) composite served as an integrated electrode. The PAM-based gel electrolyte can fix all of the components of ESC and make good contact with the collectors. In this study, a CPP composite was synthesized by wood fractionation, PAM/lithium chloride impregnation, polyaniline (PANI) electrochemical deposition, and edge cutting. The as-prepared ESC showed a wide potential window (up to 1.6 V), and high specific surface area capacitance (up to 1.73 mF/cm^2^). The present construction strategy of ESC provides a new possibility for the development of naturally sourced and high-performance multifunctional energy storage devices.

## 2. Materials and Methods

### 2.1. Materials

Balsa wood (Ochroma lagopus Swartz) with dimensions of 15 × 15 × 1 mm was purchased from Zhuhai Dechi Technology Co., Ltd. (Zhuhai, China). Aniline (ANI, AR), ammonium persulfate (APS, AR), acrylamide (AAm, AR), sodium chlorite (NaClO_2_, AR), and lithium chloride (LiCl, AR) were provided by Shanghai Aladdin Bio-Chem Technology Co., Ltd. Acetic acid (CH_3_COOH, AR, ≥99.5%) was purchased from Chengdu Cologne Chemical Co., Ltd. (Chengdu, China). Potassium hydroxide (KOH, AR) was purchased from Chengdu Jinshan Chemical Reagent Co., Ltd. (Chengdu, China). *N*,*N*-methylenebis-(acrylamide) (MBA, AR) was purchased from Tianjin Damao Chemical Reagent Co., Ltd. (Tianjin, China). Sulfuric acid (H_2_SO_4_, AR, 98%) was purchased from Lianjiang Ailian Chemical Reagent Co. Ltd. (Zhanjiang, China). Indium tin oxide (ITO) conducting glass with dimensions of 10 × 20 × 1.1 mm was purchased from South China Xiangcheng Technology Co., Ltd. (Shenzhen, China). Anhydrous ethanol (EtOH, AR, ≥99.7%) was obtained from Tianjin Fuyu Fine Chemical Co., Ltd. (Tianjin, China). All reagents were used as received.

### 2.2. Fractionation, Impregnation, and Polymerization Processes

Balsa wood samples (2 g) with dimensions of 15 × 15 × 1 mm^3^ were immersed in 50 mL 2.5 M aqueous KOH solution at room temperature for 12 h. Then, the mixture was refluxed for 3 h at 90 °C with magnetic stirring to remove most of the hemicellulose and part of the lignin. Next, the sample was rinsed repeatedly with deionized water until the pH was equal to 7. The treated samples were put into a 150-mL conical flask, and 65 mL deionized water, 0.5 mL glacial acetic acid, and 0.7 g sodium chlorite were successively added to the flask. The mixture was heated at 75 °C for 1 h, and another 0.5 mL of glacial acetic acid and 0.7 g of sodium chlorite were subsequently added. The above process was repeated three times to obtain white, purified wood samples. The as-prepared samples were rinsed repeatedly with deionized water until the pH equaled 7 and were stored in anhydrous ethanol to obtain a white cellulose network block.

### 2.3. Preparation of Cellulose Network/Polyacrylamide (CN/PAM) Conductive Hydrogel

First, 8 g acrylamide monomer, 0.1 g ammonium persulfate, and 0.08 g N, N-methylene diacrylamide were successively added into 12 mL 6 M LiCl solution at 0 °C, stirred evenly, and then stored in an environment below 5 °C for later use. The cellulose network was impregnated in a polyacrylamide hydrogel solution, then placed in a freeze dryer, and kept in the vacuum for 15 min to allow the hydrogel solution to fill the cellulose network structure. The vacuum impregnation process was repeated three times. Finally, the cellulose network and polyacrylamide hydrogel solution were put into the oven at 50 °C to solidify for 2 h. After the hydrogel was fully solidified, the samples of the cellulose network (CN)/PAM conductive hydrogel were taken out of the petri dishes and stored in a sealed bag for later use.

### 2.4. Preparation of Cellulose Network/Polyacrylamide Hydrogel/PANI (CPP) Composites

PANI was electrodeposited on CN/PAM hydrogel with cyclic voltammetry in a three-electrode system. CN/PAM samples with a conductive area of 10 × 10 mm were used as the working electrodes. A platinum electrode was used as counter electrode, and an Ag/AgCl electrode as a reference electrode. The electrolyte contained a mixture of 0.5 mol/L H_2_SO_4_ and 0.35 mol/L aniline monomer. The potential window of electrodeposition was −0.2 to 0.8 V, and the scanning rate was 50 mV/s. CPP composite hydrogels with different electrochemical properties were prepared by controlling the number of electrodeposition cycles.

### 2.5. Electrochemical Test of CPP Electrode

In the three-electrode system, CPP composite hydrogel (10 × 10 mm) was used as the working electrode, the platinum electrode as the counter electrode, and the Ag/AgCl electrode as the reference electrode. The electrolyte was 1 M H_2_SO_4_ aqueous solution. The electrochemical properties of different CPP composite hydrogels were compared, and the CPP composite hydrogels with the best comprehensive properties were selected to be assembled into supercapacitors.

### 2.6. Assembly of Symmetric ESC Based on CPP Composite Hydrogel

ITO conductive glass was washed with water and ethanol by ultrasonication for 15 min and then stored in anhydrous ethanol for later use. The four edges of the square block of CPP composite hydrogel were cut off to prevent short-circuiting. Then, the blocks were placed in the middle of two pieces of ITO glass to form an electrochromic supercapacitor with a sandwich structure. Then, PVA film was used to encapsulate the whole device for electrochemical performance determination.

### 2.7. Characterizations

The wood, cellulose network, and CPP samples were characterized with a scanning electron microscope (SEM) using a Hitachi S-3400N SEM (Tokyo, Japan) with an acceleration voltage of 5 kV. The Fourier transform infrared (FTIR) spectra of the wood, cellulose network, PANI, and CPP samples were measured with a Nicolet iS 50 FTIR instrument (Thermo Fisher Scientific Corp., Waltham, MA, USA). The spectra of each sample were recorded from 400 to 4000 cm^−1^ with 32 scans at a resolution of 4 cm^−1^. The ultraviolet–visible (UV−vis) spectra of electrode materials and devices were measured via a PerkinElmer Lambda 950 UV−vis spectroscopy (Waltham, MA, USA) with the wavelength of 250–800 nm. A three-electrode system was employed to investigate the electrochemical properties of the samples electrodes in 1 M H_2_SO_4_ electrolyte at room temperature. A silver-silver chloride (Ag/AgCl) electrode and Pt electrode were used as the reference and counter electrodes, respectively. The electrochemical properties of electrodes were tested by cyclic voltammetry (CV), galvanostatic charge–discharge (GCD) and electrochemical impedance spectroscopy (EIS) measurements on a Corrtest CS 310H electrochemical workstation (Wuhan, China).

## 3. Results and Discussion

### 3.1. Preparation and Morphology of CPP Composite Hydrogel

**Figure 1:**
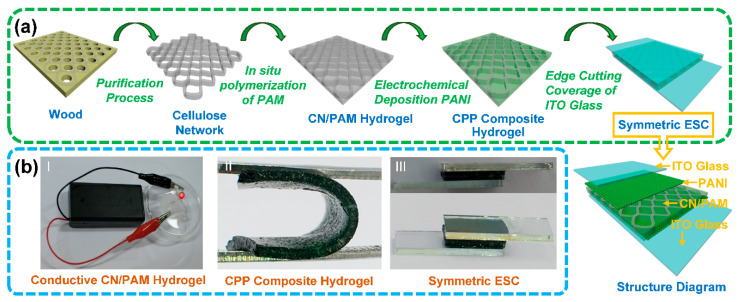
Construction procedures and digital photographs of the cellulose network/polyacrylamide/polyaniline (CPP) composite hydrogel and the corresponding electrochromic supercapacitors (ESCs): (**a**) schematic diagram of the preparation process of the symmetric ESC and its structure diagram, (**b**) digital photographs that show the conductivity of cellular network (CN)/polyacrylamide (PAM) conductive hydrogel (I), the flexibility of the CPP composite hydrogel (II), and the symmetric ESC (III).

Figure 1 summarizes the preparation process of the CPP composite hydrogel and the structure of symmetric ESCs. The CPP composite was constructed by impregnating PAM conductive hydrogel in the cellulose network and the electrochemical deposition of PANI, before cutting the four sides of the CPP composite hydrogel (Figure 1a). ITO conductive glass was introduced as a collector/transparent conductive layer and was assembled into an integrated ESC (Figure 1a). CN/PAM hydrogel exhibited promising conductivity and enabled the illumination of light-emitting diodes when connecting to the circuit (Figure 1b). The CPP composite hydrogel showed excellent flexibility, and the symmetric ESC was assembled by directly sandwiching it between two pieces of ITO glass (Figure 1b).

**Figure 2:**
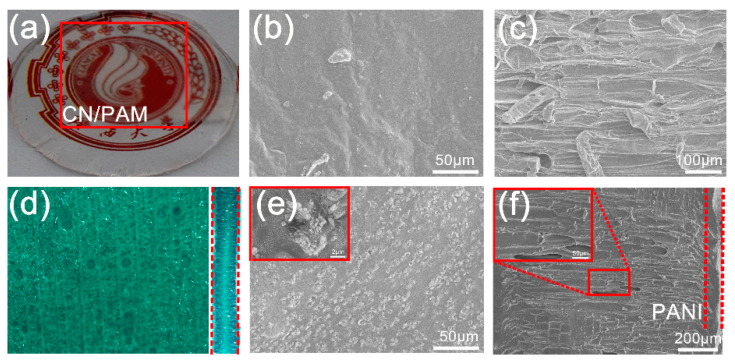
The morphology of CN/PAM and CPP composite hydrogel. (**a**) Digital photograph of CN/PAM; (**b**) SEM image of CN/PAM (cross section); (**c**) SEM image of CN/PAM (longitudinal section); (**d**) digital photograph of the cross (left) and longitudinal (right) sections of the CPP composite hydrogel prepared with 60 cycles; (**e**) SEM images of the CPP composite hydrogel (cross section); (**f**) SEM images of the CPP composite hydrogel (longitudinal section).

**Figure 3:**
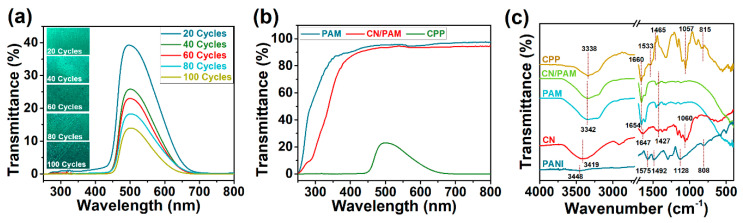
The optical and structural characterizations of the products. (**a**) UV–vis spectra of CPP composite hydrogels prepared with different electrodeposition cycles (inset: digital photographs of CPP samples with different cycles); (**b**) UV–vis spectra of PAM, CN/PAM, and CPP composite hydrogel (60 cycles); (**c**) FTIR spectra of CN, polyaniline (PANI), PAM, CN/PAM, and CPP composite hydrogel.

Figure 2 shows the morphology of the products during the preparation process of the CPP composite hydrogel. The naturally porous structure of the cellulose network makes it an excellent substrate for the construction of electrode materials and energy storage devices [12]. It has been reported that the interaction between conductive PAM hydrogel and cellulose can improve the transparency, mechanical properties, and electrical properties [18]. In this study, the mixture of PAM and LiCl first penetrated the cellulose network to provide good conductivity for PANI electrochemical polymerization. As shown in Figure 2a, PAM can enter into the internal structure of the cellulose network, endowing the obtained CN/PAM hydrogel with high transparency. SEM results and the digital photographs also confirmed that PAM penetrated the porous structure of the cellulose network (Figure 2b,c). The prepared CN/PAM composite was employed as an anode, and the in situ deposition of PANI was conducted through the electrochemical oxidation polymerization of aniline [19]. A large amount of green PANI was deposited on the cross section of the CPP composite hydrogel (Figure 2d). Interestingly, the PANI distribution on the longitudinal section of the composite showed a gradual decrease from the surface to the interior, exhibiting a layered structure (Figure 2d). Furthermore, a large number of PANI particles were deposited on the surface of the sample with a thickness of about 100 µm (Figure 2e,f). In addition, the deposition amount of PANI can be regulated by adjusting the reaction time of the electrochemical polymerization, resulting in different colors and transparencies in the sample (Figure 3a). Therefore, the CPP composite hydrogel provided an integrated structure for the construction of composite electrode materials and corresponding ESCs.

### 3.2. Optical Properties of CPP Composite Hydrogel

Figure 3a shows the digital photographs and the UV–vis spectra of CPP composite hydrogels prepared with different electrodeposition cycles. As the number of electrodeposition cycles increased, the color of the CPP composite hydrogel gradually changes from light green to dark green (Figure 3a), and the transmittance at 500 nm decreases from 39.3% to 14.0% when running between 20 cycles and 100 cycles. Ultimately, 60 electrodeposition cycles were chosen as the optimal condition, which balanced the transparency and the PANI deposition amount and enabled the three-layer structure of the CPP hydrogel (Figure 2d). Figure 3b compared the UV–vis spectra of PAM, CN/PAM, and CPP composite hydrogel (60 cycles). Both PAM and CN/PAM showed ultrahigh transmittance of more than 90% in the visible region, which indicated that PAM permeated into the microstructure of the cellulose network and filled the porous structure. The PANI deposition led to a great decrease in the transmittance and an obvious color change.

### 3.3. Characterizations of the CPP Composite Hydrogel

The interaction of the cellulose network, PAM, and PANI in the CPP composite hydrogel was analyzed by FTIR (Figure 3c). The peak assignments for the spectra are presented in Table 1. The spectrum of the cellulose network showed high similarity with that of cellulose, indicating the effective removal of hemicellulose and lignin in the purification process [20]. The PAM spectrum was basically consistent with that of CN/PAM, and its combination with the cellulose network led to a red shift in the -OH stretching vibration band, indicating that the introduction of the cellulose network weakened the hydrogen bonding [17]. In addition, as the characteristic peak of the cellulose network was not obviously highlighted in the spectrum of CN/PAM, this indicated that PAM completely entered the porous structure of the cellulose network, which was consistent with the SEM results. In the PANI spectrum, the intensity of the absorption band at 1472 cm^−^^1^ was slightly higher than that at 1565 cm^−^^1^, revealing that the as-prepared PANI contained more benzene ring units than quinone ring units and exhibited an approximate intermediate oxidation state [21,22]. Most of the characteristic peaks of the cellulose network, PAM, and PANI appeared in the spectrum of the CPP composite hydrogel, and the minor shifts in the peaks confirmed the interaction between the cellulose network and PANI. In particular, the decreased intensity and red shift in the -OH stretching vibration band showed the formation of new hydrogen bonds between the three components of the CPP hydrogel [23,24,25]. In addition, the characteristic peaks of the quinone ring and benzene ring at 1533 and 1465 cm^−^^1^ also showed a decreased intensity, and moved to a low-wavenumber region. The increased ratio of the peak intensity of the quinone ring to that of the benzene ring confirmed the high quinone ring content of the CPP composite hydrogel, which led to the excellent electrochemical performance of the composite [26]. Furthermore, characteristic peaks at 1057 and 815 cm^−^^1^ indicated the presence of π-electron delocalization in the CPP composite hydrogel caused by the hydrogen bonding between the cellulose network and PANI. The cellulose network can also provide a three-dimensional network structure that facilitates π-electron transport between PANI molecular chains, thus improving the electron/ion transport and electrochemical properties of the CPP composite hydrogel [24].

### 3.4. Electrochemical Properties of the CPP Composite Electrodes

**Figure 4:**
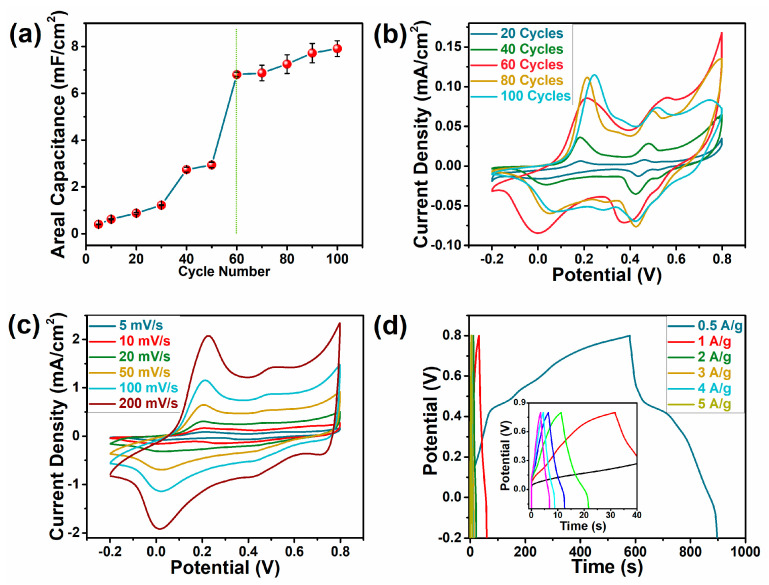
Electrochemical properties of CPP composite hydrogels with different deposition cycles. (**a**) The effect of the deposition cycle on the areal capacitance of CPP composite hydrogel; (**b**) CV curve of CPP composite hydrogels with different deposition cycles at 5 mV/s; (**c**) CV curve of the hydrogel prepared with 60 deposition cycles (CPP-60) at 5–200 mV/s; (**d**) galvanostatic charge–discharge (GCD) curve of CPP-60 at 0.5–5 A/g.

PANI was electrodeposited on CN/PAM conductive hydrogel by an electrochemical polymerization method to form the CPP composite hydrogel. During the electrochemical deposition process, more deposition cycles can lead to more PANI deposition, thus resulting in better electrochemical properties of the as-prepared CPP composite hydrogel. This was verified by our comparisons of the electrochemical properties of CPP composite hydrogels prepared with different deposition cycles (Figure 4a,b). As the number of deposition cycles increased, the areal capacitance of the resultant CPP composite hydrogel, which was attributed to the redox reaction of PANI, kept increasing. The increase in the specific capacitance of the resultant CPP composite hydrogel tended to slow down when the number of deposition cycles was 60. The CV curves of the CPP electrode materials at a scanning rate of 5 mV/s showed similar redox peaks for PANI, which became more obvious and showed typical pseudo-capacitive behavior with the increase in the deposition cycles [11,27]. Therefore, considering the transmittance and areal capacitance of CPP materials with different deposition cycles comprehensively, the hydrogel prepared with 60 deposition cycles (CPP-60) was adopted for further study.

Figure 4c presents the CV curves of CPP-60 composite hydrogel under various scanning rates in the range of 5–200 mV/s. It can be seen that the electrode redox peaks gradually widened with the increase in the scanning rate, which was mainly attributed to the different diffusion rates of H^+^ under the various scanning rates. The low scanning rate provided enough time for the diffusion of H^+^, allowing H^+^ to fully diffuse into the PANI molecular chains to complete the doping and dedoping processes [27,28]. On the contrary, a high scanning rate would lead to the insufficient redox reaction of PANI [8]. The GCD curves of CPP-60 at 0.5–5 A/g showed a typical triangle shape, which revealed the rapid charge and discharge of the electrode material with limited IR drop (Figure 4d). The Coulombic efficiency was about 100%; this could be attributed to the porous structure of the CPP composite, which contributed to the fast doping/dedoping of H^+^ [24].

### 3.5. Electrochemical and Electrochromic Properties of ESCs

**Figure 5:**
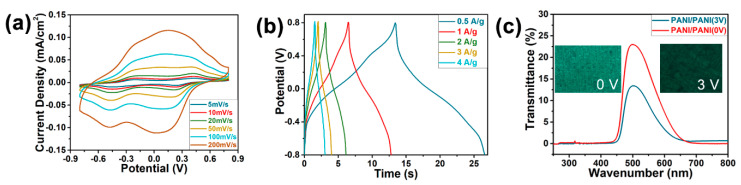
The electrochemical and electrochromic properties of the symmetric ESCs. (**a**) CV curves for the symmetric ESC at 5–200 mV/s; (**b**) GCD curves for the symmetric ESCs at 0.5–5 A/g; (**c**) UV–vis spectra and digital photographs for the symmetric ESC before and after electrification.

After the above optimization, the CPP-60 composite hydrogel was employed as the electrode material for the symmetric ESC. Figure 5 shows the electrochemical and electrochromic properties of the symmetric ESCs based on CPP composite hydrogels. CV curves under different scan rates (Figure 5a) indicated that the symmetric ESC exhibited an obvious capacitance characteristic [28]. The CV curve at 5 mV/s showed obvious redox peaks for PANI with a wide voltage window of 1.6 V, which can yield an areal capacitance of 1.73 mF/cm^2^ and an energy density of 0.62 µWh/cm^2^ at a power density of 7.03 µW/cm^2^. In addition, the GCD curves of the symmetric ESC under different current densities (Figure 5b) presented symmetric triangular shapes, which also indicated that the supercapacitor had an outstanding capacitance characteristic. The gravimetric capacitance can reach 8.22 F/g at 0.5 A/g, and the coulomb efficiency under different current densities was about 100%.

Considering the large thickness of the present ESC, a high potential of 3.0 V was applied on the device to evaluate its electrochromic performance. Figure 5c shows the UV–vis spectra and digital photographs for the symmetric ESC before and after electrification at 3.0 V. The symmetric ESC exhibited the bleached state before the electrification, and achieved a transmittance of 23.0% at 500 nm. After the electrification at 3 V, the device changed to the colored state, and the color of the positive electrode changed from light green to dark green, leading to a reduced transmittance of 13.4% at 500 nm. The transmittance decrease of 9.6% was mainly due to the color change in PANI caused by the redox reaction during the electrification process [8].

**Figure 6:**
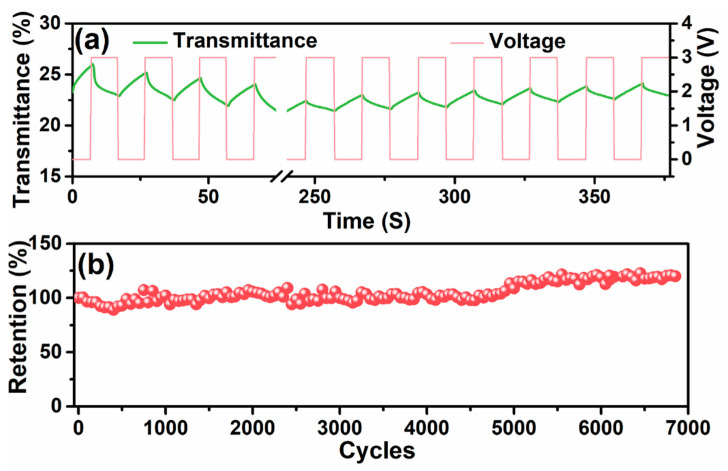
The cycling performance of the ESC device. (**a**) The transmittance-time profile of the device at 500 nm under square potential input between 0.0 V and 3.0 V at regular intervals of 10 s; (**b**) the capacitance retention of the device in the charge–discharge cycles at 1 A/g.

The cycling performance of the present ESC device was evaluated by the coloration/discoloration cycles and the charge–discharge cycles (Figure 6). The device showed a periodic transmittance change under the square potential input between 0.0 V and 3.0 V at regular intervals of 10 s (Figure 6a), indicating the promising cyclic electrochromic properties of the ESC. However, it should be noted that the transmittance change under the periodic voltage was less than that under the stable potential input of 3.0 V. The large thickness of the electrochromic layer hindered the effective transmission of lithium ions and influenced the electrochromic performance, so the device failed to fully color or discolor under the periodic potential [29]. The charge–discharge cycling performance of the ESC device was also investigated by performing more than 6800 GCD cycles at a current density of 1 A/g (Figure 6b). The ESC device showed a promising capacitance retention of more than 100% after 6850 cycles, indicating its excellent cycle stability. The increased capacitance during the charge–discharge cycles should be mainly attributed to the integrated structure of the CPP composite hydrogel. The charge–discharge may continue to create ion transport channels and enhance the electrochemical properties of the ESC device [30].

This study aimed to construct an ESC device with an integrated electrochromic layer (i.e., CPP composite hydrogel), providing a new methodology for the facile and rapid construction of ESC devices. It should be noted that the present ESC device showed a much lower transmittance change than that of the reported ESCs (up to 40%) [31]. This was mainly caused by the huge difference in the thickness of the electrochromic layer. A micron-thick electrochromic layer has usually been employed to construct thin film ESC devices [29], while a CPP composite hydrogel with a thickness of more than 1 mm was adopted as the integrated electrochromic layer to assemble the ESC device. Meanwhile, effective PANI deposition was beneficial to the electrochemical properties of the ESC, while the increase in PANI deposition conversely decreased the transmittance of the device. All these factors showed a negative effect on the transmittance change in the ESC, and resulted in limited electrochromic behavior. Further studies will focus on reducing the thickness of the CPP composite layer and balancing the electrochemical and electrochromic properties of the ESC device.

## 4. Conclusions

In this study, CPP composite hydrogels with electrochromic properties were successfully prepared based on a wood cellulose network through vacuum impregnation and electrochemical deposition, and this enabled the fabrication of symmetric ESCs. The as-prepared ESCs exhibited excellent electrochemical properties with a wide electrochemical window of 1.6 V, a high areal capacitance of 1.73 mF/cm^2^, and an energy density of 0.62 µW h/cm^2^ at a power density of 7.03 µW/cm^2^. The ESC device also showed a transmittance reduction of 9.6% at 500 nm after the electrification at 3 V, and periodic transmittance change can be achieved under the square potential input between 0.0 V and 3.0 V at regular intervals of 10 s. The biomass-derived nature of CPP composite hydrogels makes them a cheap, renewable candidate for the electrode materials of electrochromic devices, which are expected to promote the development of sustainable multifunctional energy storage devices.

## Figures and Tables

**Table 1:** Peak assignments in the FTIR spectra [16,20,21,22,23,24,25,26,27].

Sample	Wavenumber (cm^−1^)	Assignment
Cellulose Network	3342	-OH stretching vibration
	1647	C=O stretching vibration
	1427	-CH_2_ bending vibration
	1060	-OCH stretching vibration; C-O-C stretching vibration
PANI	3448	-OH stretching vibration
	1575	C=C stretching vibration of the quinone ring
	1492	C=C stretching vibration of the benzene ring
	1128	C-H in-plane bending vibration
	808	C-H out-of-plane bending vibration
PAM	3342	-OH stretching vibration
	1654	C=O vibration
CPP	1533	C=C stretching vibration of the quinone ring
	1465	C=C stretching vibration of the benzene ring
